# Supplementary figures and images for: Measuring cancer driving force of chromosomal aberrations through multi-layer Boolean implication networks
Source: PLoS One. 2024 Apr 9;19(4):e0301591. doi: 10.1371/journal.pone.0301591 (PMC11003681; doi:10.1371/journal.pone.0301591)

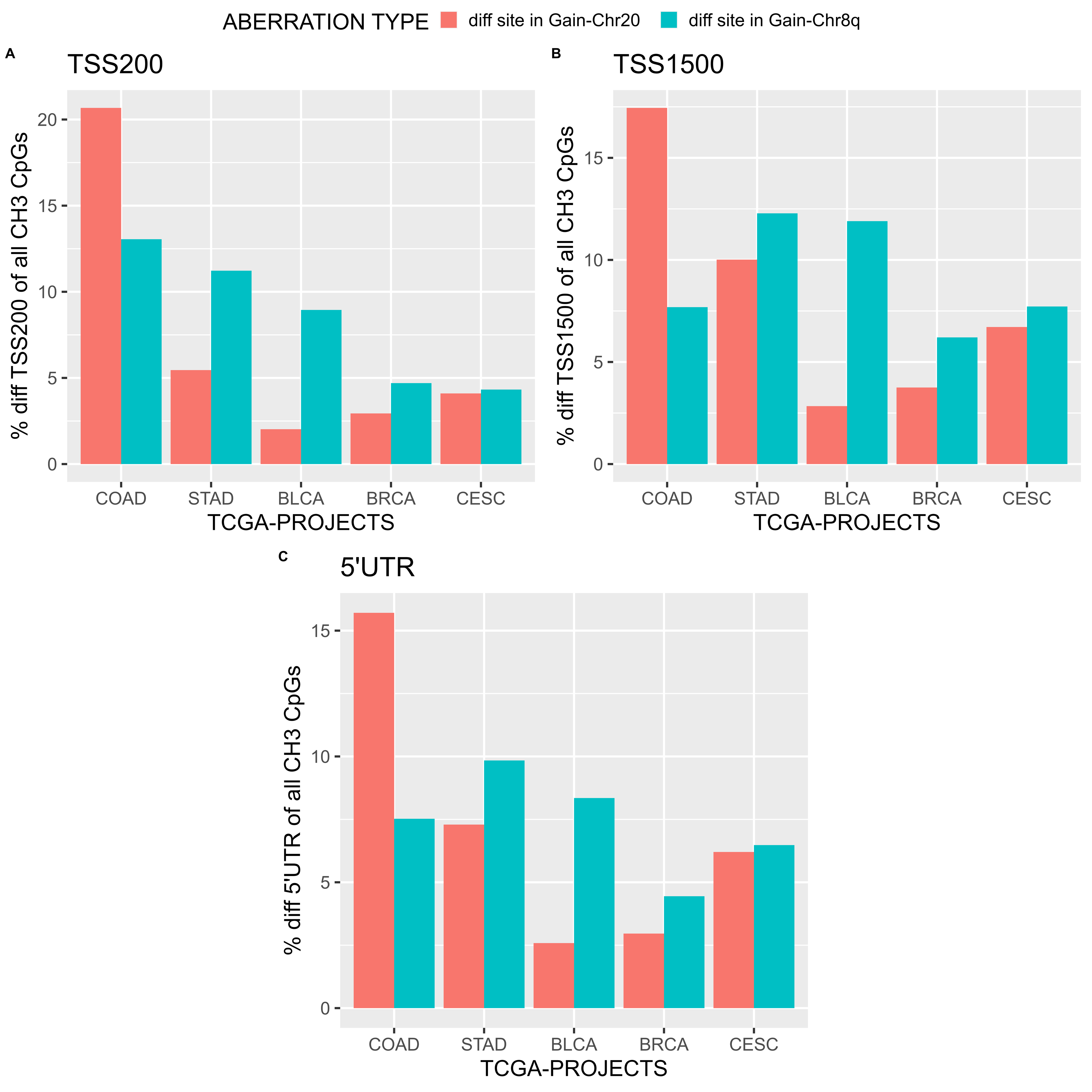

Supplement: S1 Fig — Percentage of TSS200 (A), TSS1500 (B) and 5’UTR(C) differential CpG sites in COAD, STAD, BLCA, BRCA and CESC. (TIF) [file pone.0301591.s001.tif]

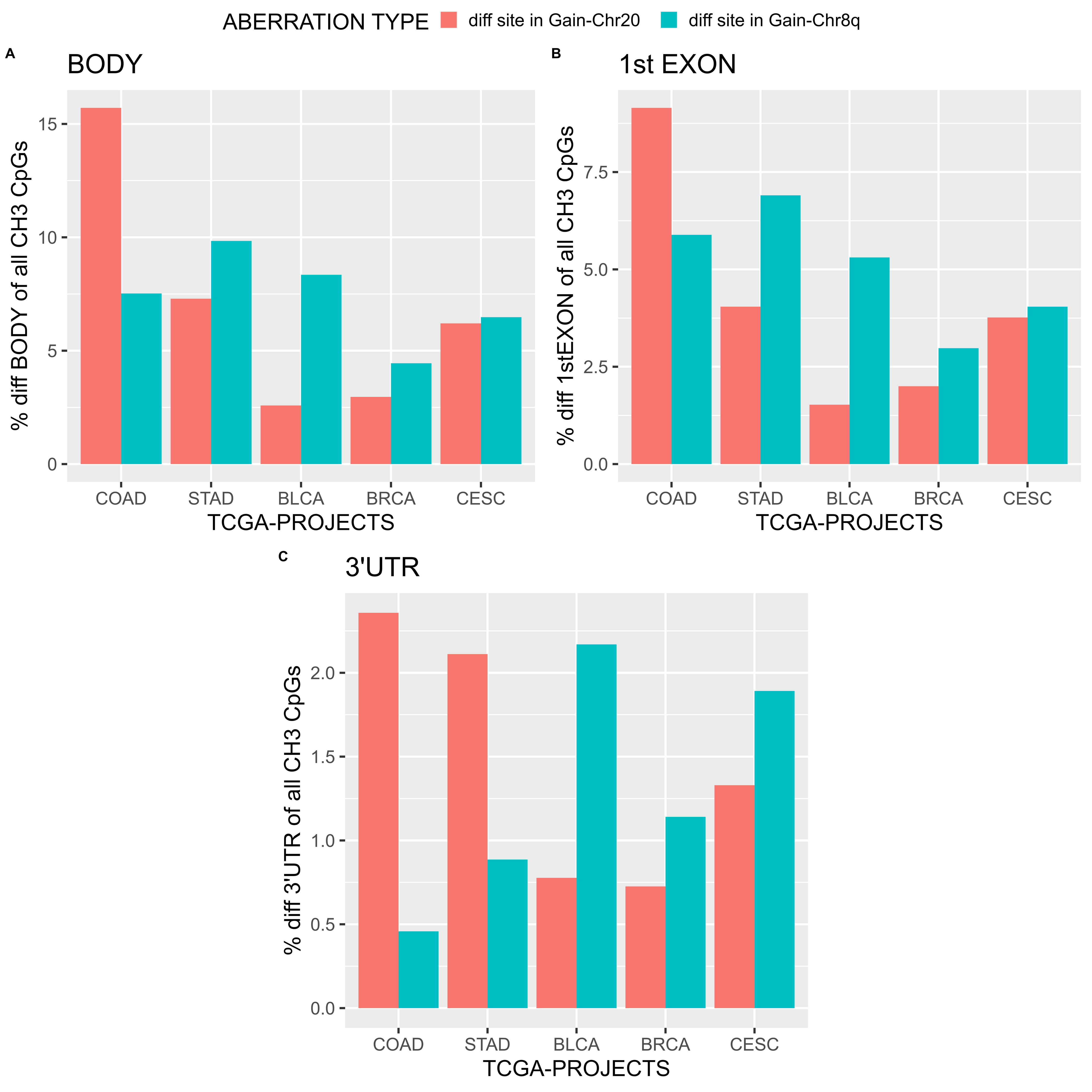

Supplement: S2 Fig — Percentage of Body (A), 1st Exon (B) and 3’UTR(C) differential CpG sites in COAD, STAD, BLCA, BRCA and CESC. (TIF) [file pone.0301591.s002.tif]
