# Supplementary material for: Measuring cancer driving force of chromosomal aberrations through multi-layer Boolean implication networks
Source: PLoS One. 2024 Apr 9;19(4):e0301591. doi: 10.1371/journal.pone.0301591 (PMC11003681; doi:10.1371/journal.pone.0301591)
Supplement: S1 Appendix — (DOCX) [file pone.0301591.s006.docx]

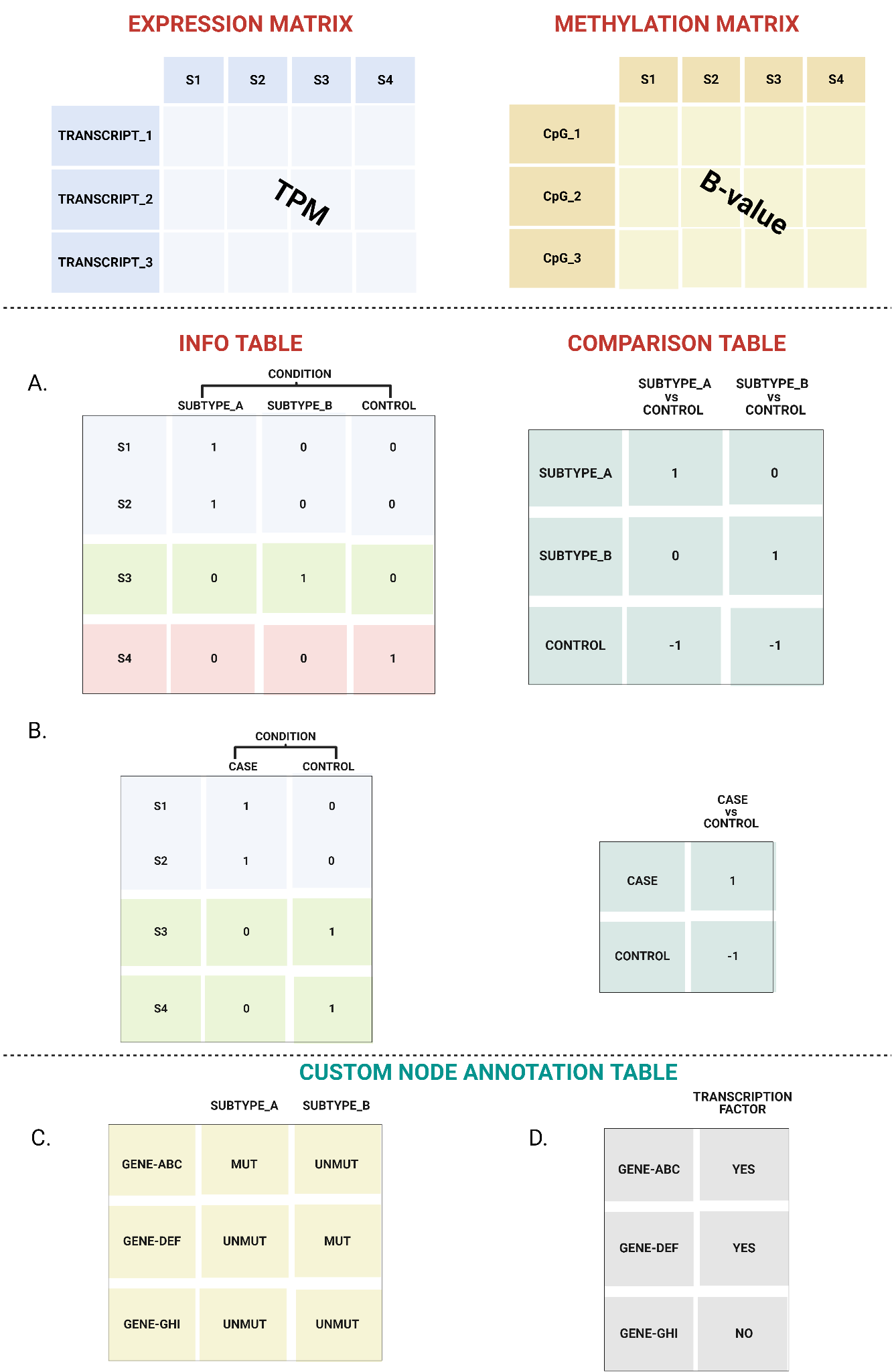


The Figure shows an example of the structure of the input files taken by COMBO. The expression matrix includes the TPM (transcripts per million) for each transcript (row) in each sample (column). The samples are differentiated from each other by a unique sample code. The methylation matrix reports the 𝛽-values and it is organized as the previously described expression matrix, except for the rows which indicate the CpG islands. The info table is used by COMBO to identify the sample groups. The unique sample identifiers are reported in the rows. Each column is binary and reports if the sample belongs (1) or not (0) to a specific condition. Finally, the comparison table is structured as the model matrix used by the R package limma, in order to establish the groups to compare. The user can choose to perform a case-control study between different types or subtypes of a tumor respect to a control group, or simply between cancer/diseased group and healthy group. In the analysis can be inserted an additional group of samples which will act as control. In the Figure are reported two different input table examples. Panel A shows the situation in which the user wants to compare two different cancer subtypes referring to a control (i.e., solid normal tissue). In this case, the control group will be used only for differential analysis. Two different multi-layer networks, one for each subtype will be generated as results of COMBO. The node annotation about the differential expression or methylation analysis will be referred to the control group. On the other hand, panel B shows the simple comparison between disease and control. The result will be a single multi-layer for the case group. The user can insert a list of optional tables in order to enrich the node annotations in the resulting multilayer and improve the specificity of the querying step. The annotations can be group-specific or general. The panel C reports an example of group-specific annotations. COMBO will insert the annotation if the node is included in the multi-layer network representing the specific condition. For example, if the node representing the gene ABC is included in the multi-layer network generated for the SUBTYPE_A, then the “MUT” annotation will be inserted. Otherwise, the annotation “UNMUT” will be added to the node DEF in the multi-layer network representing the SUBTYPE_B condition. The custom node annotation table generated as reported in panel D, will be added to the nodes independently to the condition.
