# Supplementary material for: Measuring cancer driving force of chromosomal aberrations through multi-layer Boolean implication networks
Source: PLoS One. 2024 Apr 9;19(4):e0301591. doi: 10.1371/journal.pone.0301591 (PMC11003681; doi:10.1371/journal.pone.0301591)
Supplement: Attachment — Submitted filename: response_to_reviewers.pdf [file pone.0301591.s008.pdf]

RESPONSE TO REVIEWERS

PONE-D-23-20487

Measuring Cancer Driving Force Of Chromosomal Aberrations Through Multi-layer Boolean Implication Networks.

Review Comments to the Author

Please use the space provided to explain your answers to the questions above. You may also include additional comments for the author, including concerns about dual publication, research ethics, or publication ethics. (Please upload your review as an attachment if it exceeds 20,000 characters)

Reviewer #1:

Materials and methods:

Pipeline design:

- **It would be helpful to consider providing more information about the specific input files described in [15]. Additionally, mentioning the purpose or relevance of these input files would enhance the understanding of the pipeline's setup.**

-RESPONSE: We thank the referee for this advice, which helps us clarify the input preparation of our pipeline. We added some information about input files in the “Pipeline design” paragraph (2.1):”...*The initial matrices include the unique sample identifiers in the column and the transcripts or CpG sites in the rows for expression and methylation matrices, respectively. The info table reports the list of unique identifiers for each sample and the group belonging to. COMBO exploits this table to split the input matrices for each condition. The comparison table is structured as the model matrix used in the limma R package [23]. It is used in order to identify the conditions to compare. The number of columns in the comparison table equals the number of multilayer networks generated in the final step. In addition, an optional input table reporting custom node annotations which will be used in the querying step. These table must be formatted with ENSEMBL ID in the rows and the group belonging to as columns. A detailed explanation of the input file is reported in the S1 Appendix.*”. In addition we generated a Supplementary file with a more detailed description of the input along with a new explanatory figure.

- **It would be beneficial to include a brief overview or rationale of the overall objective and significance of the pipeline, suggesting how it might address existing challenges in cancer research or omics data analysis. This could help readers understand the motivation behind the pipeline and its potential impact in the field.**

-RESPONSE: As requested by the reviewer, we introduced the following sentences at the end of the 2.1 paragraph: “*COMBO strength is in its versatility: many aspects and research areas within the field (i.e., different cancer subtypes or general pathologies) can be evaluated and*

compared in order to discriminate pathology specific elements. In addition, the integration of different omics data helps to identify complex interactions, patterns and correlations. Finally, the innovative querying step of COMBO reduces the dimensionality of the dataset, selecting the essential elements. Despite the advantages in using COMBO, it is relatively slowly because of Boolean Implications identification is a time consuming task.".

Case study:

• It might be useful to provide more details about the Reactome enrichment analysis using the ReactomePA R package. A brief explanation of how the enrichment analysis was conducted, including the specific settings or thresholds used to identify enriched pathways, could be suggested. Additionally, mentioning the significance level or multiple testing correction method employed in the enrichment analysis would improve the reproducibility and transparency of the case study, allowing other researchers to replicate the analysis and interpret the results more effectively.

-RESPONSE: In response to this comment, detailed information about the setting used for the enrichment analysis were added at the end of 2.2 paragraph: "*Briefly, the enrichment analysis was conducted separately for each condition, selecting a p-value cutoff equals to 0.05 with the Benjamini-Hochberg correction. Then the enriched resulting dataframe were combined, removing the common genes in order to generate a final image including only the genes contained in the GAIN-Chr20 network.*".

Data analysis:

• The authors introduce the term "NCDI" (Normalized Chromosomal Index). To ensure clarity, it might be helpful to move the definition of this acronym to a location where it is more relevant to the context.

-RESPONSE: Thank you for the comment. During the review phase we decided to remove the NCDI due to the fluctuating results and re-elaborate the analysis evaluating the percentage of differential nodes (genes or CpG sites) located on the n-th chromosome (Chr20 or Chr8q) with respect to the total number of nodes in the mRNA-Expression or CH_3-Methylation layer, respectively, to explain the cancer driver effect of chromosomal aberration.

Results:

Boolean Implication networks of transcriptomic data:

• Suggest discussing the significance of results: The results show NCDI values for differential nodes in different cancer types. Including an interpretation of the implications or significance of these findings and relating them to the research questions or hypotheses being addressed would enhance the discussion section.

-RESPONSE: With regarding this question, the differential analysis has been revised and the differential nodes in different cancer types have been expressed as percentage of total nodes in mRNA-Expression network layer or methylation layer for GAIN-Chr20 or GAIN-Chr8q for all the analyzed TCGA projects. Moreover, the differential nodes (genes) located on Chr20 or Chr8q have been expressed as percentage of total nodes in mRNA-Expression network layer for GAIN-Chr20 or GAIN-Chr8q for all the analyzed TCGA projects. At this regards the

following subparagraph have been introduced: "*When we analyzed the list of specific genes participating to the Boolean implication networks, focusing on mRNA-Expression layer, of GAIN-Chr20 by functional enrichment analysis, the enriched pathways across the TCGA project studied did not show any overlapping. This result suggests that strong cancer driver properties of GAIN-Chr20 might be mediated by completely different multigene-cooperation patterns in the context of different cancer types. On the other hand, analyzing the nodes representing genes located on Chr20 that are linked in the multilayer, we observed a set of genes and respective enriched pathways in common across the projects. Indeed, some genes correlated with Chr20 amplification (i.e. MYBL2, AURKA and EIF2S2) are included in all the subnetworks selected by Neo4j query. Consequently, the pathways mainly enriched are similar across the projects. Probably, the effect of GAIN-Chr20 is not circumscribed to the chromosome altered, but the aberration affects in turn different pathways and genes located in different chromosomes. For these reasons, we observed a redundancy of information selecting only genes located on Chr20, but not analyzing the whole mRNA-Expression network layer.*"

• Suggest providing more details on the statistical analysis: The Pearson correlation index and p-value mentioned in the text should be further elaborated upon. Providing more details about the statistical methods used for this analysis and how they interpret the correlation between NCDI ratio and the frequency of GAIN-Chr20 could enhance the readers' understanding.

-RESPONSE: We agree with the reviewer's assessment and due to the partial congruence of some results during the revision phase have been removed from the paper the analysis based on NCDI parameter.

Differential nodes in Boolean implication networks and functional pathway enrichment:

• Suggest including a more thorough interpretation of the results of the functional pathway enrichment analysis for different TCGA projects. Elaborating on the biological implications of the identified enriched pathways and their relevance to the research objectives or the known biology of the studied tumors with chromosome-20 gain would make the discussion more insightful.

-RESPONSE: as requested by the reviewer we rephrased different sentences in the paragraph "Discussion".

• For Figure 6, suggest expanding the caption to describe the axes, labels, and how the dots were generated (e.g., significance, gene ratio). This additional information will help readers better understand the information conveyed in the figure without having to refer to additional materials or methods sections.

- RESPONSE: Thank you for this suggestion. The Figure 6 caption was revised as follow: "*Dotplot showing the top 10 enriched pathways using differential nodes in GAIN-Chr20 groups for COAD, STAD, BRCA and CESC. The x-axis shows the enriched pathways (p-value = 0.05). BLCA results are not significant and consequently excluded from the plot. The results*

are reported for each project separating differential nodes in GAIN-Chr20 belonging to expression (RNA) and methylation (CH3) layers (as indicated in y-axis). The dot colour indicates the adjusted p-value as listed in the color bar in order to notify the significance of the enriched term. The dot size represents the gene ratio.”.

Discussion:

• Consider discussing potential limitations of the study and the COMBO pipeline to provide a balanced perspective. Mentioning any data-related or analytical constraints that might impact the interpretation of the results would strengthen the discussion.

- RESPONSE: According to this useful comment, throughout the manuscript, we have extended the discussion section inserting the limitations of our study and in general of COMBO.

• Suggest providing insights into potential future research directions based on the findings. Discussing how the results from this study could inform future studies or guide further investigations in cancer genomics and computational biology would add value to the discussion section.

- RESPONSE: We think this is an excellent suggestion. In response to this comment (and comment of reviewer #2) we inserted in the discussion the following sentence: “*Indeed, the multilayer-method (COMBO) could be exploited to filter the crucial elements in different chromosomal aberration which collaborate to execute the cancer driving effect. The identification of key pathways and specific genes which specifically act in a phenotype, could improve the identification of molecular target.* ”.

Reviewer #2:

The manuscript does an excellent job of exploring the application of the COMBO (COMBining multi-Omics) bioinformatics pipeline for the integration and analysis of multi-layer networks combining various forms of omics data. The work demonstrates the strength of COMBO in identifying critical genes in specific diseases and evaluating the interaction between DNA methylation and transcription. The authors' demonstration of COMBO's utility in analyzing multiple TCGA PanCancer Atlas project datasets effectively underscores its potential in cancer research.
However, I would like to propose a few minor suggestions for consideration:

• Discussion of Results: While the authors presented a robust argument for the use of COMBO in the analysis of numerical chromosomal aberrations like GAIN-Chr20, further clarity would be beneficial. Specifically, expanding on the methodology and rationale behind the selection of these aberrations and the consequences of these aberrations on different cancers could be insightful.

- RESPONSE: We appreciate the reviewer’s comment.

• Additional Use Cases: Given the demonstrated success of the COMBO pipeline in filtering vast RNA-seq and methylation data, it would be interesting to see it applied to other complex datasets. Exploring this further could provide stronger validation of the approach's versatility and robustness.

- RESPONSE: Thank you for pointing this out. We had planned to analyze the effect of a different chromosome aberration using COMBO in a new manuscript, with the aim to overcome the COMBO limitations. However, we decided to insert some preliminary results of chromosome 8q in order to confirm, as suggested by the reviewers, the versatility of COMBO.

• Clarification of Terminology: While the manuscript is largely well-written, some terminologies (such as Boolean implication networks) might not be familiar to all readers. I recommend including brief explanations or references for these terms to make the paper more accessible to a broader audience.

- RESPONSE: We have added the suggested explanation to the manuscript on the 2.1 paragraph using the following sentence: "*The boolean implication between the expression and methylation levels of genes are extracted in the form of "if-then" relationships. All the potential pairs of values of genes in each sample are plotted in a scatter plot which is divided, based on the StepMiner threshold, into four quadrants "low-low", "low-high", "high-low", "high-high". For each pair of genes the algorithm checks which quadrant is significantly sparsely populated with points compared to the other quadrants. In the scatter plot each point represents the expression value of the pair of genes for a specific sample. Based on the empty quadrant in the scatter plot, the algorithm detects six types of Boolean implications. The Boolean relationships can be symmetric or asymmetric. Symmetric relationships include the equivalent type, which corresponds to highly positively correlated genes, or the opposite type, which corresponds to the highly negatively correlated genes. The asymmetric ones correspond to every sparse quadrant and are divided into low - low, high - low, low - high, and high - high.*"

• Future Work: The authors suggest that the information contained in gene expression Boolean implication networks can provide a quantitative estimate of the cancer-driving force of GAIN-Chr20. This proposition is exciting and potentially groundbreaking. It would be valuable if the authors could discuss more on how this method can be further exploited in future research, perhaps by providing some potential scenarios or implications.

- RESPONSE: As asked by reviewer #1, we inserted the future directions on the discussion paragraph.

• In conclusion, I found the manuscript to be of high quality, presenting a compelling case for the use of COMBO in the analysis of multi-omics data and its potential impact on cancer research. These minor suggestions are intended to polish the already excellent work and help the authors in disseminating their findings to the widest possible audience.

-RESPONSE: We would like to thank the reviewer for this positive evaluation.

Reviewer #3:

In this work by Cosentini et al., the authors introduce a novel pipeline that helps to get a systems view of multi-omics data by analyzing heterogeneous multilayered networks constructed from transcriptomic, epigenetic, and metabolic databases. They also evaluate their pipeline (COMBO) in various TCGA cancer datasets. As a result of their analysis, they identify broad gain in chromosome 20 on different cancers as a driving force. Though their pipeline is novel and could be a useful tool in medical research, there is a lack of clarity in their pipeline which might affect the results significantly and might change the conclusion. If the authors clarify the following details and also explain the consequence of altered consideration, this pipeline could be a good tool for medical research. I recommended a revision of the work.

Specific points:

1. During the Boolean network constriction, the authors have used different values of s-statistics ranging from 2-4, which might affect the strength of the network very much.

-RESPONSE: Thank you for pointing this out. The reviewer is correct, and we have decided to even the s-statistic across the five TCGA projects. Consequently, we selected the same s-threshold for expression data or methylation data. We described in detail in the text (2.2 paragraph "Case study") the s-statistic used inserting a new table with the information (Table 2). In addition, we computed false discovery rate on 20 different randomly permuting gene expression dataset (S2 File).

2. The authors have not described the p-statistics cutoff they used during network construction.

-RESPONSE: : We have added the suggested content to the manuscript on "Case study" paragraph using the following sentence: "*A p-value cutoff equals to 0.01 was used to select the statistically significative Boolean Implications. The default values were chosen for the remaining parameters.* "

3. It is not clear if they used a Boolean clustered network or simply use all the genes as separate nodes. If they used each gene as a separate node, then the size of the network and edge distribution will change the result significantly. If they use the clustered network, they haven't described the cutoffs used in the cluster formation of different Boolean relations.

-RESPONSE: Thank you for raising this question. In this manuscript we didn't generate Boolean cluster networks. BooleanNet was used to identify Boolean implication between transcripts or CpG probes. Then, the final multilayer network is constituted by genes as separate nodes.

4. The terminology they have used is not consistent throughout the paper. Which could be confusing for the reader.

-RESPONSE: Thank you for pointing this out. We have corrected inconsistent terms.

5. The authors haven't described the statistical cutoff used and their logic during the identification of the interlayer edges from KEGG pathways.

-RESPONSE: As required by the referee the following logic has been adopted "*The interlayer edges are inserted between two nodes representing the same gene but located in different layers. For example, if gene A is located on both mRNA and CH3 layer, an inter-layer edge is added in order to link the two nodes. In this way, we are linking the expression and methylation information of the same gene. Similar situation occurs if the geneA is located on both mRNA and MP layer*".

-RESPONSE: We are afraid that we do not understand the referee's criticism completely. We will try to respond to the best of our understanding. The interlayer edges are inserted between two nodes representing the same gene but located in different layers. For example, if geneA is located on both mRNA and CH3 layer, an inter-layer edge is added in order to link the two nodes. In this way, we are linking the expression and methylation information of the same gene. Similar situation occurs if the geneA is located on both mRNA and MP layer.

6. The authors evaluate their pipeline by identifying chromosome 20 over various cancers. I believe the analysis of the gain or loss of at least another chromosome is required to properly evaluate the pipeline.

-RESPONSE: Thank you for this observation. As answered for reviewer #2, we reported a new case study using chromosome 8q amplification.
