# Supplementary material for: Measuring cancer driving force of chromosomal aberrations through multi-layer Boolean implication networks
Source: PLoS One. 2024 Apr 9;19(4):e0301591. doi: 10.1371/journal.pone.0301591 (PMC11003681; doi:10.1371/journal.pone.0301591)
Supplement: Attachment — Submitted filename: Review_report_HZ.docx [file pone.0301591.s007.docx]

Materials and methods:

Pipeline design:

- It would be helpful to consider providing more information about the specific input files described in [15]. Additionally, mentioning the purpose or relevance of these input files would enhance the understanding of the pipeline's setup.
- It would be beneficial to include a brief overview or rationale of the overall objective and significance of the pipeline, suggesting how it might address existing challenges in cancer research or omics data analysis. This could help readers understand the motivation behind the pipeline and its potential impact in the field.

Case study:

- It might be useful to provide more details about the Reactome enrichment analysis using the ReactomePA R package. A brief explanation of how the enrichment analysis was conducted, including the specific settings or thresholds used to identify enriched pathways, could be suggested. Additionally, mentioning the significance level or multiple testing correction method employed in the enrichment analysis would improve the reproducibility and transparency of the case study, allowing other researchers to replicate the analysis and interpret the results more effectively.

Data analysis:

- The authors introduce the term "NCDI" (Normalized Chromosomal Index). To ensure clarity, it might be helpful to move the definition of this acronym to a location where it is more relevant to the context.

Results:

Boolean Implication networks of transcriptomic data:

- Suggest discussing the significance of results: The results show NCDI values for differential nodes in different cancer types. Including an interpretation of the implications or significance of these findings and relating them to the research questions or hypotheses being addressed would enhance the discussion section.
- Suggest providing more details on the statistical analysis: The Pearson correlation index and p-value mentioned in the text should be further elaborated upon. Providing more details about the statistical methods used for this analysis and how they interpret the correlation between NCDI ratio and the frequency of GAIN-Chr20 could enhance the readers' understanding.

Differential nodes in Boolean implication networks and functional pathway enrichment:

- Suggest including a more thorough interpretation of the results of the functional pathway enrichment analysis for different TCGA projects. Elaborating on the biological implications of the identified enriched pathways and their relevance to the research objectives or the known biology of the studied tumors with chromosome-20 gain would make the discussion more insightful.
- For Figure 6, suggest expanding the caption to describe the axes, labels, and how the dots were generated (e.g., significance, gene ratio). This additional information will help readers better understand the information conveyed in the figure without having to refer to additional materials or methods sections.

Discussion:

- Consider discussing potential limitations of the study and the COMBO pipeline to provide a balanced perspective. Mentioning any data-related or analytical constraints that might impact the interpretation of the results would strengthen the discussion.
- Suggest providing insights into potential future research directions based on the findings. Discussing how the results from this study could inform future studies or guide further investigations in cancer genomics and computational biology would add value to the discussion section.
